# Ion-Pair Speciation in Aqueous Alkali Fluorides from Nuclear Magnetic Resonance of ^19^F

**DOI:** 10.1021/acs.jpcb.5c08394

**Published:** 2026-04-09

**Authors:** Małgorzata Musiał, Samantha Miller, Christopher L. Suiter, Heidi Klem, Eugene Paulechka, Kathleen A. Schwarz, Jason Widegren, Demian Riccardi

**Affiliations:** † Department of Physics, University of Colorado, Boulder, Colorado 80309, United States; ‡ Applied Chemicals and Materials Division, 535691 National Institute of Standards and Technology, Boulder, Colorado 80305, United States; § Materials Science and Engineering Division, 10833 National Institute of Standards and Technology, Gaithersburg, Maryland 20899, United States

## Abstract

We demonstrate that a combination of multiscale modeling and experimental ^19^F NMR can be used to examine the molecular details of how ion-pair speciation changes with temperature and molality. Excellent agreement with experimental ^19^F NMR chemical shifts is attained through the pairing of the theoretical ion-pair chemical shift profile with speciation populations estimated with molecular dynamics simulations and thermodynamic equilibrium constants. Clear periodic trends down Group I metal cations show that the ion-pair chemical shift profile is dominated by the shielding effects of pairing-induced dehydration for smaller cations (Li^+^ and Na^+^) that are overwhelmed by short-range deshielding effects for heavier cations (K^+^, Rb^+^, and Cs^+^). The experimental ^19^F NMR chemical shift trends with increasing temperature and molality are connected explicitly to changes in ion-pair populations relative to the free-ion state. This work provides a general approach to model ion pairing and solvation within the framework of NMR, which has implications for understanding ion-pairing phenomena in various systems.

## Graphical abstract

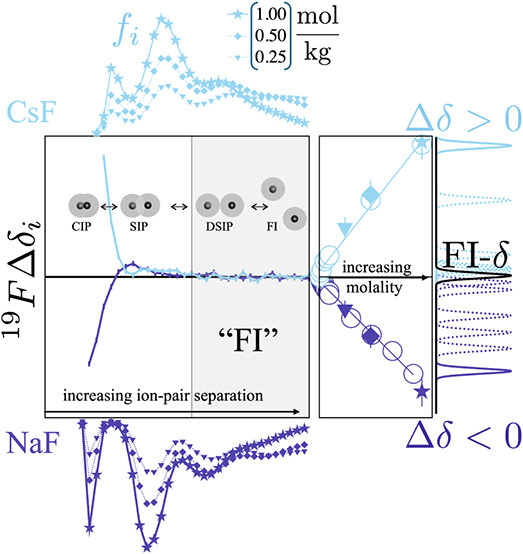

## Introduction

Aqueous electrolyte solutions are complex systems in which ion–water and ion–ion interactions set the electrostatic stage for a wide range of hydrated processes that are crucial for life and industry.^1–5^ Beyond their fundamental relevance in solution chemistry, ion-pairing phenomena play a decisive role in driving selectivity and reactivity in modern catalysis and electrochemistry.^6–8^ The nature of these interactions is both complex and subtle—hydrated free ions exist in equilibrium with paired ions.^9^ While decades of theoretical studies have provided a molecular framework for understanding ion hydration^10–12^ and ion pairing,^13–15^ researchers face a significant challenge in validating molecular simulation potentials^13,16^ due to the limited availability of high-quality experimental data associated with ion-pairing equilibria. Experimental methods such as Raman, dielectric, and acoustic spectroscopies have failed to fully translate spectra into a detailed molecular picture of ion pairing, even in simple alkali halide solutions.^17–20^

Nuclear Magnetic Resonance (NMR) spectroscopy has long been recognized as a sensitive probe of ionic environments in solution. An early investigation by Deverell et al.^21^ reported that the ^19^F chemical shift in aqueous alkali fluorides is relatively insensitive to the identity of the cation, salt concentration, and temperature, suggesting that solvation dominates the observed signal. Similarly, Tong et al.^22^ systematically examined the concentration dependence of the ^19^F chemical shift in aqueous alkali fluoride solutions, emphasizing the primary role of solvation. These foundational studies were limited to bulk interpretations that could not distinguish between the role of solvation and ion-pairing interactions.

Recently, we used NMR spectroscopy to separate the effects of free-ion hydration and ion pairing in NaF solutions.^23^ To interpret these experiments, we developed a multiscale modeling framework that combines polarizable force field molecular dynamics (MD) simulations with quantum chemical calculations of NMR shielding tensors for ion-pair-water clusters. This initial study was able to accurately capture the changes in resonance frequency with temperature for the free-ion state and a single concentration of NaF. Here, we carry out a broader application to a range of alkali metal ions (Li^+^ to Cs^+^) and concentrations, testing our methodology and deepening our general understanding of ion pairing in electrolyte solutions. We can now predict contact ion pair (CIP) and single-solvent-separated ion pair (SIP) populations that correspond to the experimental chemical shifts for a wide range of temperatures and concentrations.

## Experimental and Theoretical Methods

### NMR Chemical Shifts

NMR spectroscopy measures the absolute resonance frequency (ν_abs_) absorbed by nuclei with multiple nuclear spin states due to an applied magnetic field (*B*_0_),

1 $$\nu_{\mathrm{abs}}=\frac{\gamma B_{0}}{2\pi }\left(1-\sigma \right)$$

where *B*_0_ is the scalar magnitude of the external field, *γ* is the nuclear gyromagnetic ratio, and *σ* is the shielding constant. Resonance frequencies are given in Hz units, and the chemical shift (*δ*) values (in ppm units) are readily calculated as,

2 $$\delta =\frac{\nu_{\mathrm{abs}}-\nu_{\mathrm{ref}}}{\nu_{\mathrm{ref}}}\times 10^{6}\;\mathrm{ppm}$$

where *ν*_ref_ is the absolute resonance frequency of the reference signal. In our recent study, we used Hz units to allow comparisons across different nuclei, ^19^F and ^23^Na. In the present work, we focus on ^19^F, which allows us to use the ppm scale. Plugging eq 1 into eq 2,

3 $$\delta =\sigma_{\mathrm{ref}}-\sigma$$

relates the chemical shift directly to the isotropic form of the NMR shielding tensor, which we calculate using quantum chemistry.

In this study, we change the reference (*σ*_ref_) to isolate the chemical shifts for different types of interactions. We use the physically motivated terms “shield” and “deshield” to describe decreases and increases, respectively, in the chemical shift with respect to the reference state.

### Sample Preparation

Stock solutions of XF salts were prepared by weighing a known mass of each salt before dissolving in ultrapure water. The source and purity of the chemicals used are shown in Table S1. Serial dilutions were then used to prepare additional molalities. The molalities used for each XF salt in this study are shown in Table S2. For NMR analysis, these solutions were added to 5 mm O.D. thin-walled glass NMR tubes. Next, a 2 mm O.D. coaxial insert containing a 1% solution of DSS-*d*_6_ in D_2_O was inserted into the glass NMR tube. This arrangement allowed for deuterium field locking and chemical shift referencing without influencing solvation or ion pairing. With the coaxial tube arrangement, the chemical shift reference, DSS, is in a different compartment than the sample solution; thus, differences in bulk magnetic susceptibility (BMS) in the two compartments must be considered.^23^ To determine the magnitude of the BMS effect on referencing, we performed experiments in which the locations of the sample solution and reference solution were switched. These control experiments were done for the highest concentrations of NaF and CsF: 0.9 mol·kg^–1^ NaF in H_2_O and 1.0 mol·kg^–1^ CsF in H_2_O. These two solutions are at the extremes of the current study, so any changes due to BMS effects would be relatively easy to discern. One spectrum was collected with the salt solution in the outer tube and the DSS solution in the coaxial inner tube. Then the position of the solutions was switched (the DSS solution in the outer tube and the salt solution in the coaxial inner tube), and a second spectrum was collected. For both NaF and CsF, the difference in ^19^F *δ* for the two spectra was <0.01 ppm, which is well within our measurement uncertainty. Consequently, no correction was made for BMS effects, and no BMS term is included in the working equations.

We also verified that fluoride-glass reactions do not affect our results. Control ^19^F NMR measurements were performed in plastic tubes for representative concentrations across the series. The resulting chemical shifts were identical, within experimental uncertainty (0.03 ppm), to those obtained in standard thin-walled glass tubes, confirming that fluoride interaction with glass does not influence the reported data within this concentration range.

### NMR Spectroscopy

All NMR experiments were collected using a 14.1 T (600 MHz) Bruker UltraShield spectrometer operating at basic transmitter frequencies of 600.130 MHz and 564.686 MHz for ^1^H and ^19^F, respectively. A BBO probe with a *z*-gradient was used for data collection. The probe was automatically tuned and matched for each sample, nuclei, and temperature condition. Temperatures were controlled with a flow of nitrogen gas at a rate of 400 L·h^–1^. Temperatures were calibrated using 99.5% methanol-*d*_4_ and an 80% ethylene glycol/20% DMSO-*d*_6_ solution. The calibration procedures have been reported previously.^24,25^ The resulting experimental temperatures were 280.46 K, 286.70 K, 288.99 K, 297.43 K, 313.21 K, 326.8 K, 335.89 K, 342.58 K, 347.79 K, 353.27 K, 358.27 K, and 364.24 K (±0.1 K for *T* ≤ 297.43 K; ±0.22 K for *T* > 297.43 K). Due to the coaxial setup and presence of D_2_O, deuterium locking and automatic shimming were used in all cases.

For ^1^H data collection, a one-pulse experiment with a typical 90° excitation pulse width of 17.9 μs was used. The spectral width was set at 12.02 ppm and 32,768 data points were collected. The acquisition time was 2.27 s and the recycle delay was set to 3 s. Sixty-four scans were collected per experiment. The data were processed using a Fourier transformation. The data were zero-filled to 65,536 points, and 0.3 Hz of exponential line-broadening was applied. The data were phased manually, and a polynomial was used for baseline correction. The ^1^H NMR was used to determine the temperature-dependent chemical shift of DSS-*d*_6_ in D_2_O as reported by Hoffman.^26^ These values were then used to set an appropriate spectrum reference frequency for the ^19^F experiments.

For ^19^F data collection, a spin-echo sequence with two homospoil/*z*-gradient pulses was used (zggpse).^27^ This was done to eliminate a broad NMR resonance that arises from a piece of Teflon within the NMR probe, which caused baseline distortions. The 90° pulse for this sequence was 12.95 μs. The spectral width was set to 491.96 ppm. The acquisition time was 0.94 s. A recycle delay of 3 s was used. 524,288 data points were collected using 32 scans. Data were processed using a Fourier transformation. Data were zero-filled to 1,048,576 (or 1024^2^) points, and 3 Hz of exponential line broadening was applied. The data were phased manually, and a polynomial baseline correction was applied. During data processing the spectrum reference frequency obtained from calibrating the ^1^H NMR signal of DSS-*d*_6_ was used to ensure the ^19^F chemical shift signals appeared at the correct chemical shift.

Data analysis, interpretation, and uncertainty considerations for ^19^F NMR follow the protocols established by Musiał et al.^23^ In brief, NMR chemical shifts are chosen by a maximum peak intensity routine, common in most NMR analysis software. The uncertainties in sample concentration, temperature, phasing, line-broadness, peak symmetry, baseline correction, and DSS-*d*_6_ assignment are then used to evaluate an uncertainty for the ^19^F chemical shift. The value we report here as the final chemical shift is the value obtained from the maximum peak intensity routine with error bars based on an expanded (*k* = 2) uncertainty of 0.03 ppm.

### Computational Methods

The multiscale modeling framework was developed for NaF and described, in detail, in the Supporting Information of our recent study.^23^ We briefly describe the methods here for completeness with a focus on highlighting workflow additions and changes. We also direct readers to our data publication (https://data.nist.gov/od/id/mds2-3157); in addition to parameters, representative scripts, the data publication contains Jupyter Lab notebooks that analyze extensive dataframes of radial distribution functions (RDF), minimum-distance distribution functions, and chemical shifts to show how the data was analyzed. The XYZ coordinates of all ion-pair clusters along with the MDAnalysis scripts for RDF and minimum-distance distributions are also included.

Initial configurations were generated using PackMol.^28^ We used MDAnalysis for structural analysis.^29^ There were 1, 23, 45, and 90 ion pairs in a box of 5000 water molecules for biased runs of a single pair and unbiased runs at (0.25, 0.5, 1.0) mol·kg^–1^, respectively. We ran four independent seeds for each unbiased run at each temperature. The biased-run configurations were packed independently at each distance in 0.0125 nm increments from the closest distance up through 1.0 nm. The closest distance sampled were:

- LiF: 0.15 nm
- NaF: 0.20 nm
- KF: 0.25 nm
- RbF: 0.25 nm
- CsF: 0.25 nm

The biased MD simulations included a harmonic restraint with constant of 10460 kJ·mol^–1^·nm^–2^ (25 kcal·mol^–1^Å^–2^) applied to X–F separation distance.

The AMOEBA09 polarizable force field^30^ for water molecules^31^ and ions,^32^ was modified with the Thole damping parameter for fluoride reduced from 0.39 to 0.2;^33,34^ m-AMOEBA09 is used as the shorthand. GPU-accelerated Tinker-GPU^35^ was used for all simulations. Initial configurations were minimized to 4.184 kJ·mol^–1^·Å^–1^ (1.0 kcal·mol^–1^·Å^–1^). All simulations were carried out in the isothermal–isobaric ensemble (NPT) at four temperatures (280 K, 300 K, 330 K, 365 K) using a RESPA integrator with a 2 fs outer time step and a 0.5 fs inner time step 2.0 fs timesteps of the RESPA timestep integrator.^36^ Temperature was maintained using a Bussi thermostat^37^ with a 0.2 ps coupling time, and the pressure was maintained at 1.01325 bar using a Monte Carlo barostat with a relaxation time of 2.0 ps. Periodic boundary conditions were used along with a 1.2 nm cutoff for van der Waals interactions along with a long-range VDW correction (input keyword vdw-correction). Electrostatic interactions were calculated using a 0.7 nm real space cutoff; long-range electrostatics were included using the particle mesh Ewald method.^38^

An initial set of simulations for LiF, NaF, KF, and RbF, based on our earlier workflow,^23^ was run with 1 fs timesteps and a cutoff of 0.7 nm for van der Waals (VDW) interactions. We compared the RDF and minimum-distance distributions and found no significant differences between the 1 fs and 2 fs workflows, described above. Each unbiased run was well-converged after 50 ns to 75 ns of production. We also compared the ion-pair chemical shift profiles between the two sets and found they agreed very well. As a result, we combined all the chemical shift data for both sets. Unbiased production runs were saved every 10.0 ps. Biased-window production runs were saved every 5.0 ps.

Isotropic NMR shielding tensors were calculated using gauge-independent atomic orbital (GIAO) method^39,40^ as implemented in Gaussian 16 ^41^ paired with the ωB97X-D/ma-TZVP level of theory.^42,43^ The ma-TZVP basis set^44^ minimally augments the def2-TZVP basis set^45^ to provide s and p diffuse basis functions on non-hydrogenic atoms; effective core potentials were used for Rb and Cs. All quantum chemical calculations incorporated the polarization of the bulk region outside the ion-pair clusters using the integral-equation formalism polarizable continuum model,^46^ as implemented in Gaussian 16 with the keyword SCRF=PCM. The NMR chemical shift profile for ^19^F was calculated as a function of X–F separation by averaging the ^19^F isotropic NMR shielding tensors in 0.025 nm bins collected from all XF-pair water cluster geometries extracted from the biased MD trajectories; the expanded (*k* = 2) uncertainty was used for all error bars. Each cluster geometry includes both ions and all water molecules located within 0.45 nm of the cation, the anion, or the midpoint between them. The biased MD simulations and quantum chemical calculations were carried out independently for each temperature. Ion-pair configurations were extracted every 250 ps, which yielded around 100,000 XF ion-pair cluster configurations for quantum chemical calculations. The free ion (FI) region was set at 0.6 nm for all XF salts.

We model the ^19^F NMR chemical shifts due to ion pairing using a weighted sum of the ion-pair profile of the chemical shifts from the ^19^F FI-*δ* reference value,

4 $${\Delta \delta }_{\mathrm{theory}}\left(b,T\right)=\sum f_{i}\left(b,T\right){\Delta \delta }_{i}\pm \sqrt{\sum f_{i}^{2}U_{i}^{2}}$$

where *f*_*i*_(*b*,*T*) is the fraction of the species associated with a point on the NMR chemical shift profile, Δ*δ_i_* ± *U*_*i*_, and *U*_*i*_ is the expanded (*k* = 2) uncertainty. In this framework, the observed macroscopic change in chemical shift with molality is driven entirely by population reweighting. The underlying theoretical single-ion-pair chemical shift profile (Δ*δ_i_*) represents the fundamental physical interaction and is invariant with concentration. To calculate the profile, we employ biased MD simulations of a solvated single XF ion pair at multiple temperatures and restrained interionic distances; the Δ*δ*_*i*_ values are then calculated using quantum chemical methods for the extracted clusters. We assume Δ*δ*_*i*_ is temperature-independent, as supported by SI figures showing profiles overlapping, within the uncertainties, at varying temperatures (Fig. S1).

Unbiased simulations are not suitable for estimating ion-paring populations at low molality, due to convergence issues arising from the relatively small number of ions compared to the large number of water molecules. This limitation is particularly significant for LiF, which has a large binding constant and limited solubility (up to 0.05 mol·kg^–1^). To make meaningful comparisons with experimental data, we employ an alternative approach for low molality systems, using experimental equilibrium constants to estimate the fraction of CIP. As described below, we make additional modeling choices to include contributions from the SIP region. We calculate the overall shift using a two-state version of eq 4,

5 $${\Delta \delta }_{\mathrm{theory}}\left(b,T\right)=f_{\mathrm{CIP}}\left(b,T\right){\Delta \delta }_{\mathrm{CIP}}+f_{\mathrm{SIP}}{\Delta \delta }_{\mathrm{SIP}}\pm \sqrt{f_{\mathrm{CIP}}^{2}U_{\mathrm{CIP}}^{2}+f_{\mathrm{SIP}}^{2}U_{\mathrm{SIP}}^{2}}$$

where *f*_CIP_ is the fraction of CIP that is estimated from equilibrium constants using initial concentration and temperature-dependent activity coefficients;^47,48^ the Δ*δ*_CIP_ ± *U*_CIP_ and Δ*δ*_SIP_ ± *U*_SIP_ values are calculated from associated regions of the theoretical ion-pair chemical shift profile (Figure 2B, Table 1). The uncertainty values are calculated at a 95% level of confidence level (*k* = 2) for the regional selection of chemical shifts. We calculate the SIP fraction from CIP fraction and the ratio of CIP to SIP, which can be estimated using the RDF of the 0.25 mol·kg^–1^simulations of CsF and NaF (Fig. S5, Table 1); for LiF, this ratio is treated as a fitting parameter.

**Table 1:** Equilibrium Constants and Regional Chemical Shifts for ^19^F for CIP and SIP

Salt	*K*_a_ ^a^	Δ*δ*_*i*_ CIP^b^	Δ*δ*_*i*_ SIP^c^	*a*_CIP/SIP_ ^d^
LiF	1.78	–22.7 (0.5)	2.5 (0.3)	0.33
NaF	0.47	–11.7 (0.6)	1.1 (0.2)	0.12
CsF	0.07	12.9 (0.9)	1.3 (0.5)	0.16

a Equilibrium constants are taken from ref 55 at 298 K. We assume that the equilibrium constant represents the formation of CIP regardless of other ion-pair species, eq 6 Standard state 1 mol·dm^–3^.

b Mean of values within 0.025 nm of the CIP peak in the RDF (0.2 nm, 0.24 nm, 0.28 nm, 0.3 nm, 0.31 nm for LiF, NaF, KF, RbF, and CsF) and their uncertainties in parenthesis.

c Mean of values within 0.1 nm of the SIP peak in the RDF (0.35 nm, 0.44 nm, 0.46 nm, 0.48 nm, 0.49 nm for LiF, NaF, KF, RbF, and CsF) and their uncertainties in parenthesis.

d Ratio of CIP to SIP populations calculated from the RDF at 300 K. LiF values were adjusted to the best fit to the experimental shifts.

### Description of NMR Chemical Shift Symbols

To eliminate ambiguity and for convenience, we provide symbol definitions:

^19^F *δ*: The experimental value determined for low molality using the DSS signal as the reference.

FI-*δ*: The theoretical values use the mean value of the free ion *σ* (averaged over temperatures 280 K, 300 K, 330 K, 365 K) as the reference (*σ*_ref_); with this reference choice, the FI-*δ* downward trend with temperature passes through zero at ∼319 K.

^19^FΔ*δ*_*i*_: The ion-pair ^19^F NMR chemical shift evaluated at discrete cation–anion separation distances indexed by *i*. The profile is averaged over temperatures (280, 300, 330, 365 K). Before averaging the final profile, each profile, at a given temperature, is calculated referenced to the average isotropic shielding tensor (*σ*) of the free ion (FI) for that temperature. With this reference choice, the chemical shift profile of the ion-pair decays to zero at long distances (>0.6 nm).

^19^FΔ*δ*: The total chemical shift due to ion pairing reported using the FI-*δ* values as the reference. The theoretical values are calculated using eq 4.

### Using Equilibrium Constants to Calculate Regional Fractions

To estimate the fraction of contact ion pairs (CIP) in lower molality salt solutions, we rely on equilibrium constants (*K*_a_). To do this, we needed to make our own modeling choices. First, we assume that *K*_a_ corresponds to the equilibrium between the CIP population and everything else,

6 $$K_{a}=\frac{m_{\mathrm{CIP}}}{m_{\mathrm{FI}}^{2}\gamma_{\pm }^{2}}$$

where *m*_CIP_ is the molality of the CIP, *m*_FI_ is the molality of everything else—such that the initial concentration provides a convenient path to the fraction estimate using fundamental modeling from general chemistry (Initial, Change, Equilibrium); *γ*_±_ is the mean activity coefficient of the cation and anion, which must be calculated as described further below. Next, we define the ratio of CIP to SIP (*a*_CIP/SIP_) in order calculate the fraction of SIP,

7 $$f_{\mathrm{SIP}}=\frac{f_{\mathrm{CIP}}}{a_{\mathrm{CIP}/\mathrm{SIP}}}$$

The *a*_CIP/SIP_ parameter is useful as it can be estimated from radial distribution functions or used as a fitting parameter (Table 1).

### Activity Coefficient Calculations

Activity coefficients (γ_*i*_) of ions in aqueous solutions are necessary to account for deviations from ideal behavior due to ion–ion interactions and ion–solvent interactions. Several models have been derived over the years with the most popular being the Debye-Hückel model. This model has a version in which the limiting law is extended:^47,48^

8 $$\log_{10}\left(\gamma_{i}\right)=\frac{A{\left|Z_{i}\right|}^{2}\sqrt{I}}{1+Ba^{0}\sqrt{I}}-\log\left(1+0.001m_{i}M_{s}\right)+CI$$

where *a* ^0^ is the ion size parameter,^49^ *m*_*i*_ is the molality, and *I* is ionic strength of the electrolyte. For investigated salts, *I* = *m*_*i*_. *C* is the ion-interaction parameter, and *M*_S_ is the molar mass of the solvent, which in our case is H_2_O (18.015 g·mol^–1^). The last part of the equation in the limit of very low concentration is negligible (*e.g*, *C* = 0.012 kg^2^·mol^–2^ at 298.15 K, thus for our highest concentration, *CI* = 0.002 kg·mol^–1^). *A* and *B* are Debye–Hückel parameters and can be calculated for changing density:^48^

$$A\left({\mathrm{mol}}^{-\frac{1}{2}}\;{\mathrm{kg}}^{\frac{1}{2}}\right)=1.8247\times 10^{6}\left[\frac{{\left(d\left({g\;\mathrm{cm}}^{-3}\right)\right)}^{\frac{1}{2}}}{{\left(\varepsilon_{r}T\left(K\right)\right)}^{\frac{3}{2}}}\right]$$ 9

$$B\left({Å}^{-1}\;{\mathrm{mol}}^{-\frac{1}{2}}\;{\mathrm{kg}}^{\frac{1}{2}}\right)=50.2901\left[\frac{{\left(d\left({g\;\mathrm{cm}}^{-3}\right)\right)}^{\frac{1}{2}}}{{\left(\varepsilon_{r}T\left(K\right)\right)}^{\frac{1}{2}}}\right]$$ 10

Where *d* is the density of the solvent, *ε*_r_ is the dielectric constant of the solvent both of which are dependent on temperature^50,51^ . This approximation for activity coefficients is considered accurate for our concentrations <0.10.

## Results and Discussion

The hydrated free-ion (FI) state provides the reference that is used to isolate the influence of ion pairing on the NMR signal. By measuring the ^19^F NMR chemical shift of fluoride for the FI state (FI-*δ*) at low concentrations, we can determine FI-*δ* for all alkali fluoride salts (XF). The FI state is invariant to the cation, as confirmed by comparing FI-*δ* for each XF salt to that of non-associating tetramethylammonium fluoride (TMAF)^52^ across a range of temperatures (Figure 1A). The largest difference in chemical shift from TMAF was less than 0.08 ppm (inset of Figure 1A). FI-*δ* decreases with increasing temperature, a phenomenon that we investigate using theoretical calculations. Using ion-pair clusters extracted from biased MD simulations (Figure 2A), we estimate FI-*δ* from all XF clusters with ion-pair distances beyond 0.6 nm and find good agreement with experiment (Figure 1B). As shown previously using only NaF,^23^ the decrease in FI-*δ* with temperature results from the shift in the solvation populations toward smaller numbers of first-shell water molecules (Figure 1C). The strong linear correlation (∼40 ppm increase from 0 to 6 water molecules, Figure 1D) between FI-*δ* and the number of water molecules reflects the strong interactions between F^–^ and water.

**Figure 1:**
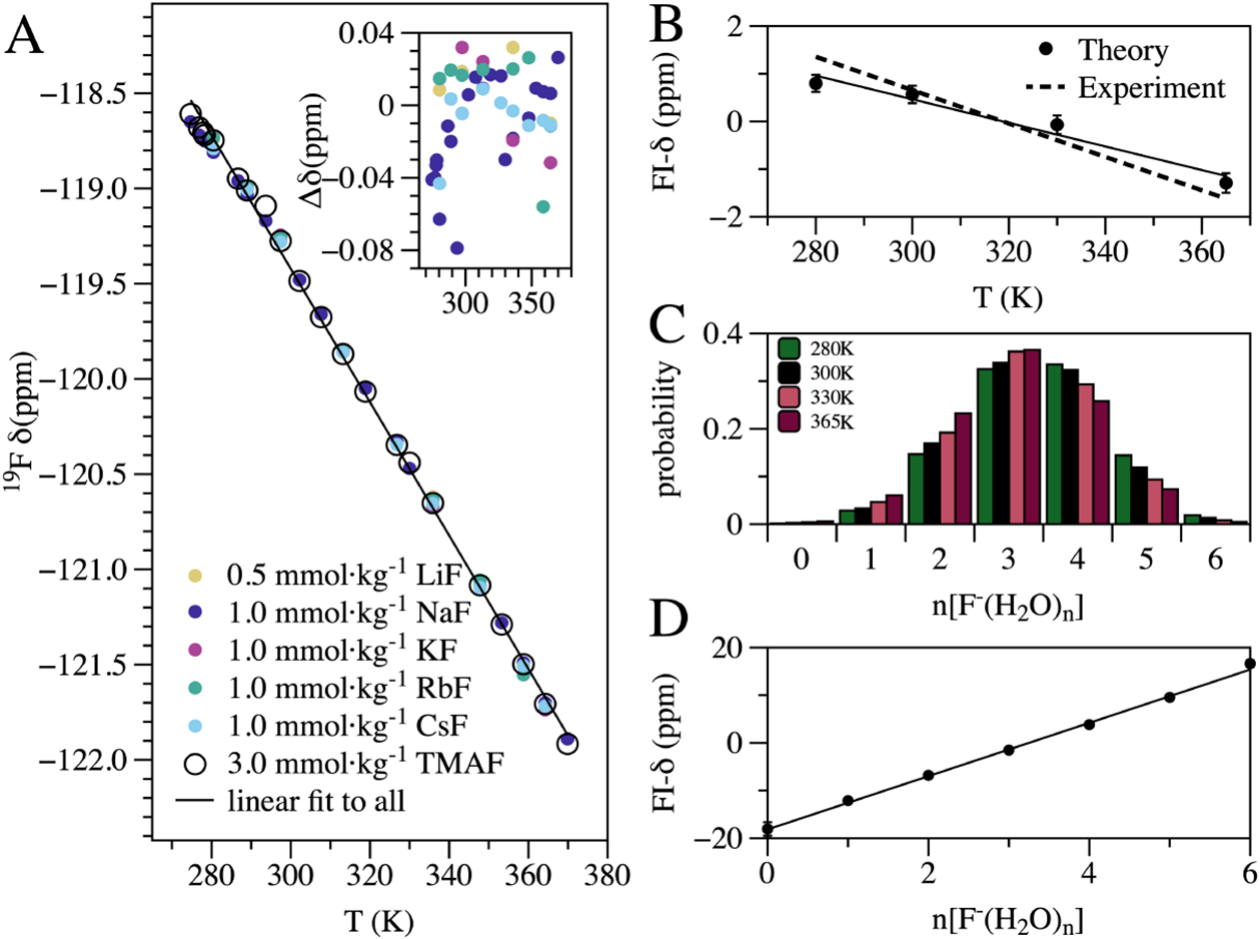
Temperature dependence of the ^19^F chemical shift for the free-ion (FI) state. (A) Experimental ^19^F chemical shifts for dilute alkali fluorides and TMAF. The error bars are within the point size. Inset: differences from TMAF reference. (B) Theoretical FI chemical shifts (referenced to their average) and a linear fit to the experimental data in (A) (dashed line). (C) Histogram of the solvation number (water molecules with a hydrogen atom within a 0.2 nm cutoff). The average number of strongly interacting waters within this cutoff is 3.5 at 300 K, whereas integrating the full F–O radial distribution function yields a total first-shell coordination number of 6.4. (D) Linear increase in theoretical ^19^F chemical shift with increasing solvation number.

The theoretical ^19^F NMR chemical shift profiles (Δ*δ* *_i_*, Figure 2B) show distinct trends with cation–anion distance. Notably, the most significant variations in the chemical shift occur over a relatively narrow spatial range of roughly 0.1 nm. This sharp transition corresponds to the region where the ions’ first hydration shells are disrupted to form a contact ion pair. Beyond 0.6 nm, the ions are fully solvent-separated, and the shift naturally returns to the free-ion baseline. Within the CIP region itself, for Li^+^ and Na^+^, the ^19^F nucleus is shielded by ∼20 ppm relative to FI-*δ*, primarily due to pairing-induced dehydration (Fig. S2). In contrast, heavier alkali metals (K^+^, Rb^+^, and Cs^+^) deshield the ^19^F nucleus. The CIP region profiles for K^+^ and Rb^+^ oscillate, while Cs^+^ consistently deshields ^19^F nucleus. For the larger cations, the shielding contributions from dehydration are overwhelmed by strong short-range deshielding in the contact region. As previously described using experimental and theoretical studies of solid-state alkali fluorides^53,54^ this deshielding is governed by electronic overlap between the interacting anion-cation pairs, an effect that increases with cation size. The Δ*δ* *_i_* values for the complete XF series reinforce our recent observation for NaF^23^ that significant NMR signal contributions arise from ion pairs beyond the CIP region. This finding suggests that the ion-pair reach of NMR may be longer still, given the low experimental uncertainties.

**Figure 2:**
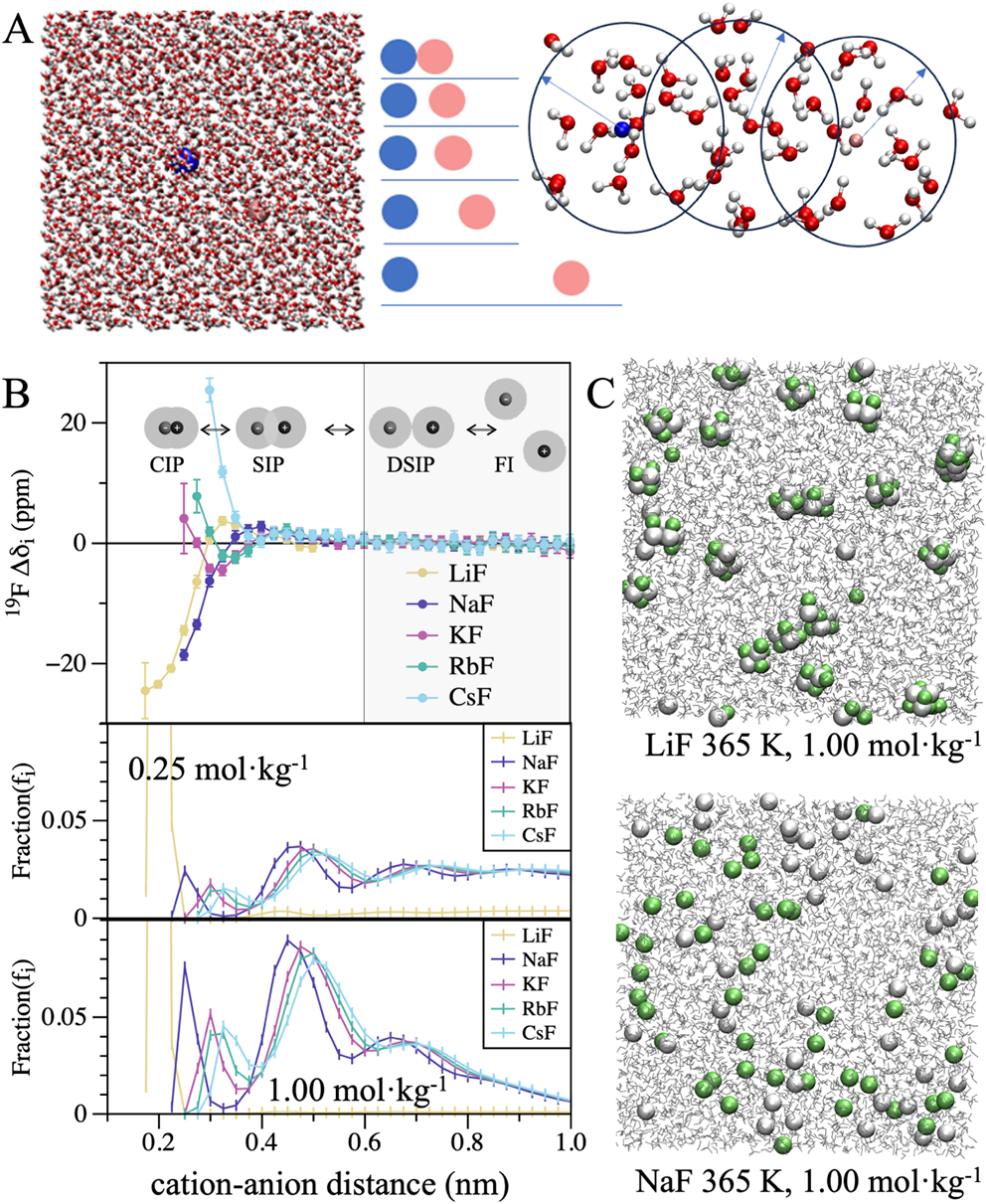
Calculation of theoretical chemical shifts and ion-pairing analysis. (A) Workflow schematic showing a single ion pair solvated in 5000 water molecules and simulated at varying interionic distances to generate clusters for quantum chemical calculations. (B) Calculated ^19^F chemical shifts vs. cation–anion distance (top) and minimum-distance distributions for XF salts at (0.25 and 1.0) mol·kg^–1^ (bottom) at 330 K. The ion-pairing states are shown with the following labels: Free ion (FI), double-solvent-separated ion pair (DSIP), single-solvent-separated ion pair (SIP), contact ion pair (CIP). The shaded region (>0.6 nm) is considered the free-ion (FI) region. (C) Representative snapshots from 1 mol·kg^–1^ trajectories at 365 K for LiF and NaF, showing different high-order clustering behavior for LiF.

We analyzed the minimum-distance distributions between ions and their counterions from unbiased MD simulations at varying molalities (0.25, 0.5, 1.0) mol·kg^–1^ to estimate ion-pairing fractions (*f*_*i*_ in eq 4). The minimum-distance distribution categorizes each ion based on its nearest counterion, unlike the ion-pair radial distribution function (RDF), which includes all ion pairs. We note that these distributions are normalized to unity over the entire simulation volume. Consequently, the visible area under the curves plotted in Figure 2B is unequal across concentrations; for lower molalities like 0.25 mol·kg^–1^, a much larger fraction of the population resides in the unplotted free-ion region (>1.0 nm). Our framework assumes that the NMR shift for a given nucleus is dominated by its nearest counterion and neglects more distant neighbors and higher-order salt clusters. For example, LiF forms extensive higher-order salt clusters at higher molalities (Figure 2C), which are not treated by our modeling framework and are also not experimentally accessible due to the limited LiF solubility. Analysis of the minimum-distance distributions revealed that increasing molality and temperature enhances the CIP population (Figure 2B and Fig. S3). The CIP populations for the non-LiF salts at the highest temperature and molality remain relatively low (15% to 20%, Fig. S4). The CIP peak shifts to longer distances and decreases with increasing cation size, as expected. Furthermore, the CIP-to-SIP ratio increases with molality (Fig. S5). These findings, combined with the ion-pair chemical shift profile (Figure 2B), imply that the ^19^F chemical shifts for NaF should decrease and those for CsF should increase with rising temperature and molality.

The experimental chemical shifts for the XF series change across the series with clear periodic trends. The chemical shifts for LiF and NaF decrease with increasing molality (Figure 3) and temperature (Figure 4), while those for CsF increase under the same conditions. In contrast, KF and RbF show more subtle changes, with a slight increase in chemical shift with increasing molality at 300 K (Figure 3A) and 330 K (Figure 3B). At 1.0 mol·kg^–1^, RbF exhibits a slight rise in chemical shift with temperature, while KF shows a slight decrease (Figure 4A). Even at ≤0.05 mol·kg^–1^, LiF displays a significant drop in chemical shift (∼1 ppm) with increasing molality at both 300 K (Figure 3A) and 330 K (Figure 3B). The chemical shift of 0.05 mol·kg^–1^ LiF drops by around 1 ppm from 280 K to 365 K (Figure 4B); for the same variables, the chemical shift for NaF is slightly negative, KF and RbF are close to zero, and CsF is slightly positive (Figure 4B). As we mention in Computational Methods section, to estimate the fraction of contact ion pairs (CIP) in lower molality salt solutions, we rely on equilibrium constants (*K*_a_). However, determining accurate ion-pairing equilibrium constants is a challenging task, and available literature values are limited to specific conditions and often rely on models with associated assumptions. For example, ref 55 reports equilibrium constants for the entire XF salt series at 298 K and 1 atm, derived from conductivity experiments using two different models: the Pitts equation and the Fuoss equation. Notably, the resulting *K*_a_ values exhibit significant variability between the two models, as illustrated by the case of CsF, where *K*_a_ = 0.07 (Pitts equation) and 0.46 (Fuoss equation) for the standard state 1 mol·dm^–3^. We found better agreement (Figure 3C) with the experimental NMR chemical shifts using the values for LiF, NaF, and CsF from the values using the Pitts equation, so we report those values in Table 1.

**Figure 3:**
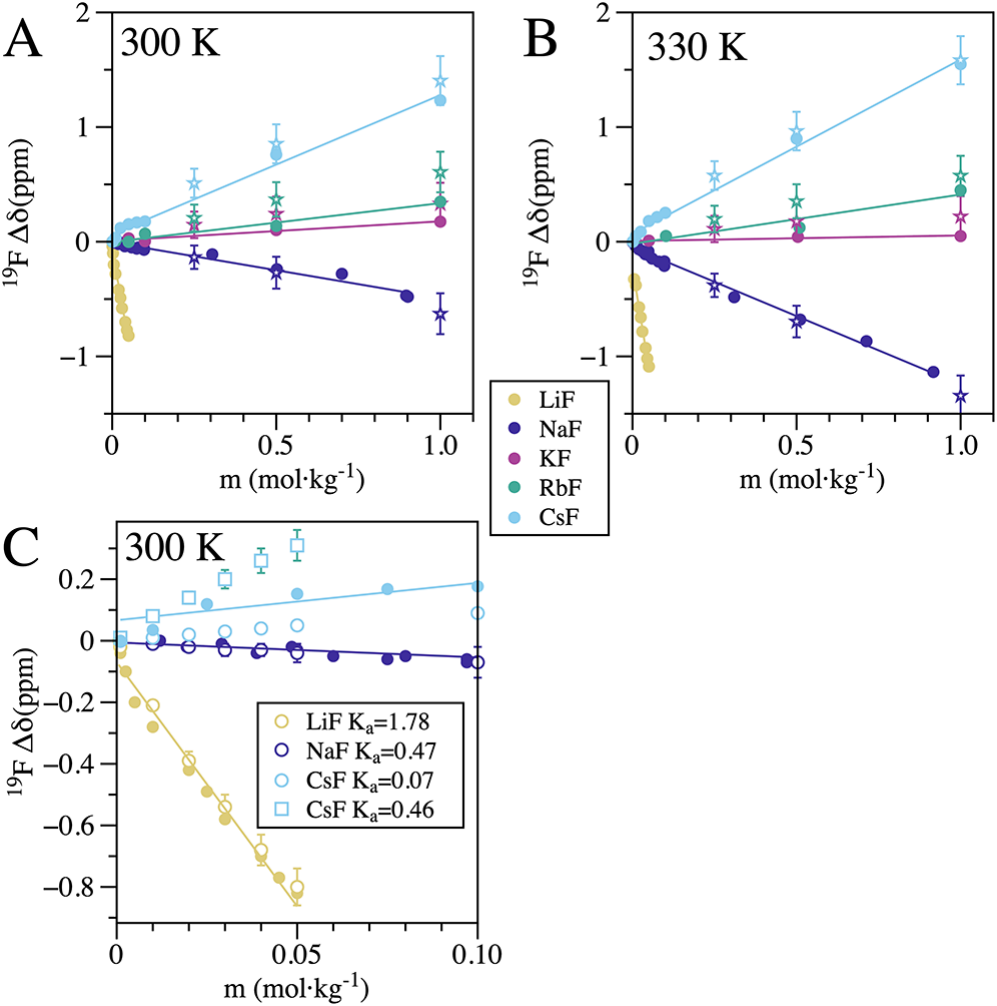
Comparison of experimental and theoretical chemical shifts relative to the FI-*δ* as a function of molality. The plots compare experimental data (filled circles) with theoretical predictions derived from MD simulation fractions (unfilled stars) and thermodynamic equilibrium constants (unfilled circles). (A) Molality dependence at ∼300 K: Experimental data collected at 297.7 K. The theoretical values are calculated using eq 4 based on the ^19^F chemical shift profiles and MD simulation populations. (B) Molality dependence at ∼330 K: Experimental data collected at 335.8 K. (C) Low molality region: Detailed view of the low concentration regime for LiF, NaF, and CsF. The theoretical values here are calculated using thermodynamic equilibrium constants (*K*_a_). For CsF, two scenarios are shown corresponding to the upper and lower bounds of the experimental equilibrium constants.

**Figure 4:**
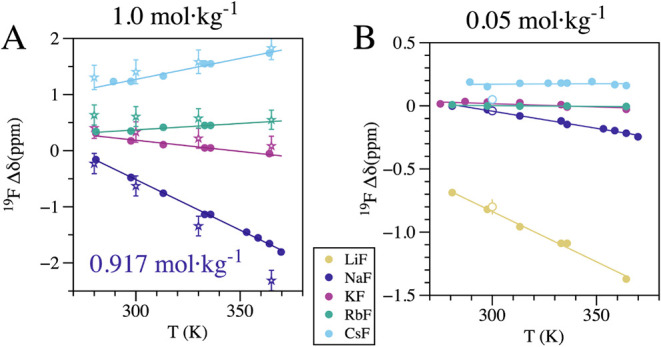
Comparison of experimental and theoretical chemical shifts relative to the FI-*δ* as a function of temperature. The plots compare experimental data (filled circles) with theoretical predictions derived from MD simulation fractions (unfilled stars) and thermodynamic equilibrium constants (unfilled circles). (A) High molality plots—comparisons are provided at 1.0 mol·kg^–1^ for the MD simulations and around 1.0 mol·kg^–1^ for other soluble salts except for NaF, which has a molality of 0.917 mol·kg^–1^. (B) Low molality plots—comparisons at 0.05 mol·kg^–1^. Theoretical values are calculated using thermodynamic equilibrium constants (*K*_a_). Only three theoretical points are included, corresponding to the temperatures where literature *K*_a_ values were available.

A detailed evaluation of equilibrium constants is beyond the scope of this study, but our approach demonstrates a proof of principle for applying our NMR chemical shift framework to lower molalities. Moreover, this application of the framework may provide a powerful tool for evaluating reported equilibrium constants. For instance, our results suggest that the *K*_a_ value of 0.46 for CsF^55^ is likely an overestimation, given the approximations and level of electronic structure theory we applied.

Our theoretical framework accurately captures the experimental trends for NaF, KF, RbF, and CsF across a wide range of molalities and temperatures, using direct unbiased MD simulations (Figure 3A,B and Figure 4A). The agreement between theory and experiment is generally excellent, with most trends reproduced. A notable exception is the subtle increase in RbF chemical shift with temperature at 1.0 mol·kg^–1^ (Figure 4A), which is not captured by the theoretical model; instead, theory predicts a flat, slight decrease like KF. Overall, the periodic trends in the ion-pair chemical shift profiles (Figure 2B), combined with the changes in ion-pair speciation populations with molality and temperature, provide a robust framework to examine the molecular details of the experimental observations. The qualitative chemical shift trends can also be understood in the context of experimental solid-state NMR data, which suggests that the trend in the chemical shift with increasing molality should ultimately approach the value for the solid,^21,56^ see SI for more details.

With these modeling choices, we successfully reproduce the low-molality trends in the chemical shifts (Figure 3C), demonstrating that the experimental trends are indicative of CIP formation. The inclusion of the SIP region in eq 5 affects the apparent CIP population. For LiF and NaF, the SIP region compensates the CIP contribution, effectively increasing the apparent CIP population; for CsF, the SIP region contributes in the same direction as the CIP region, decreasing the apparent CIP content of the chemical shift.

## Conclusions

The sensitivity of the ^19^F nucleus signal to hydration and ion pairing renders it an attractive nucleus for investigating ion pairing across a wider range of state parameters, such as higher molality, and in other systems, such as fluoride salts in different solvents containing cofactors like crown ethers. Our framework provides a general approach to model ion pairing and solvation in light of ^19^F NMR experiments; as presented here, the framework can accurately determine first-order effects of ion pairing. As demonstrated in the error analysis of our previous study^23^ for NaF, the success of this framework benefits from the robust cancellation of systematic errors in absolute shielding when evaluated as a relative shift. Ultimately, our derived ion-pair speciation estimates depend on the balance between highly accurate experimental measurements, the classical sampling potential, and the quantum chemical level of theory. Because the experimental NMR data serve as a highly accurate physical anchor, any future improvements to the accuracy of either the sampling potential or the electronic structure calculations will implicitly shift the physical implications and requirements of the other. We have demonstrated the use of equilibrium constants to model low molality conditions, and conversely, the NMR profile can be used to estimate equilibrium constants from experimental shifts. Effects such as chemical reactions or higher-order clustering will cause deviations that may be productively evaluated as perturbations to the ion-pairing reference. Future extensions of the framework will involve generalizing the ion-pairing distance coordinate to include categorical speciation, with representative structures, and population estimates.

## Supplementary Material



## Data Availability

Supplementary data, parameters, representative scripts, and comprehensive data frames are available for direct download from https://data.nist.gov/od/id/mds2-3157.

## Notes

Detailed descriptions of theoretical methods and supporting analysis of molecular dynamic simulations and theoretical NMR chemical shifts (PDF)

## ∥

Eli Lilly and Company, 600 Tech Court, Louisville, CO 80027

## ⊥

Department of Biological Sciences, Louisiana State University, Baton Rouge, LA 70803
