# Supplementary material for: Ion-Pair Speciation in Aqueous Alkali Fluorides from Nuclear Magnetic Resonance of 19F
Source: J Phys Chem B. 2026 Apr 9;130(16):4388–96. doi: 10.1021/acs.jpcb.5c08394 (PMC13112355; doi:10.1021/acs.jpcb.5c08394)
Supplement: Supplementary file 1 [file jp5c08394_si_001.pdf]

Supporting information for:

Ion-Pair Speciation in Aqueous Alkali Fluorides from Nuclear Magnetic Resonance of ^{19}F

Małgorzata Musiał[1,*], Samantha Miller[2,†], Christopher L. Suiter[2], Heidi Klem[2,††], Eugene Paulechka[2], Kathleen A. Schwarz[3], Jason Widegren[2], Demian Riccardi[2,*]

[1]Department of Physics, University of Colorado, Boulder, Colorado 80309, USA

[2]Applied Chemicals and Materials Division, National Institute of Standards and Technology, Boulder, Colorado 80305, USA

[3]Materials Science and Engineering Division, National Institute of Standards and Technology, Gaithersburg, Maryland 20899, USA

[†]Present address: Eli Lilly and Company, 600 Tech Court, Louisville, CO 80027

[††]Present address: Department of Biological Sciences, Louisiana State University, Baton Rouge, LA 70803

* Corresponding authors: malgorzata.musial@nist.gov and demian.riccardi@nist.gov

Notes:

Supplementary experimental and modeling data with extensive csv-formatted dataframes and representative scripts are provided here:

https://data.nist.gov/od/id/mds2-3157

Trade names are provided only to specify the source of information and procedures adequately and do not imply endorsement by the National Institute of Standards and Technology. Similar products by other developers may be found to work as well or better.

Supporting Information

We provide a description of NMR chemical shift symbols, methods, additional tables, and figures in support of the main text.

- **Understanding ^{19}F chemical shifts due to ion paring using experimental solid-state NMR data (Scheme S1)**
- **Table S1 -** The source and purity of chemicals used for this study
- **Table S2** - The molalities of XF solutions used in this study
- **Figure S1 -** Ion-pair NMR ^{19}F chemical shift profile for each XF salt calculated independently for all temperatures
- **Figure S2 -** Ion-pair ^{19}F NMR chemical shift profile calculated using the linear model that relates **FI-*δ*** to the number of first-shell solvent water molecules
- **Figure S3 -** Minimum-distance distributions for each XF salt as a function of distance at varying molalities (0.25 mol·kg^{-1}, 0.5 mol·kg^{-1}, and 1.0 mol·kg^{-1}) and a fixed temperature (330 K)
- **Figure S4 -** Regional populations for CIP and SIP calculated from the minimum distance distributions as a function of molality and temperature
- **Figure S5 -** Ratio of CIP to SIP populations, as a function of molality, calculated from radial and minimum distance distributions for NaF through CsF
- **Figure S6 –** Color palette

Understanding ^{19}F chemical shifts due to ion-paring using experimental solid-state NMR data

In addition to using the ion-pair chemical shift profile (Fig. 2B), chemical shift trends can be understood in the context of experimental solid-state NMR data, which suggests that the trend in the chemical shift with increasing molality should ultimately approach the value for the solid[S1,S2]. For LiF, NaF, and KF, more CIP formation is expected to decrease the ^{19}F chemical shift, while for RbF and CsF, more CIP formation should increase the chemical shift. Our results are consistent with this expectation for LiF, NaF, RbF, and CsF, but not for KF, which may be due to the similarity between the solid-state ^{19}F chemical shift and the free ion value, making it difficult to distinguish the trend. See Scheme S1.

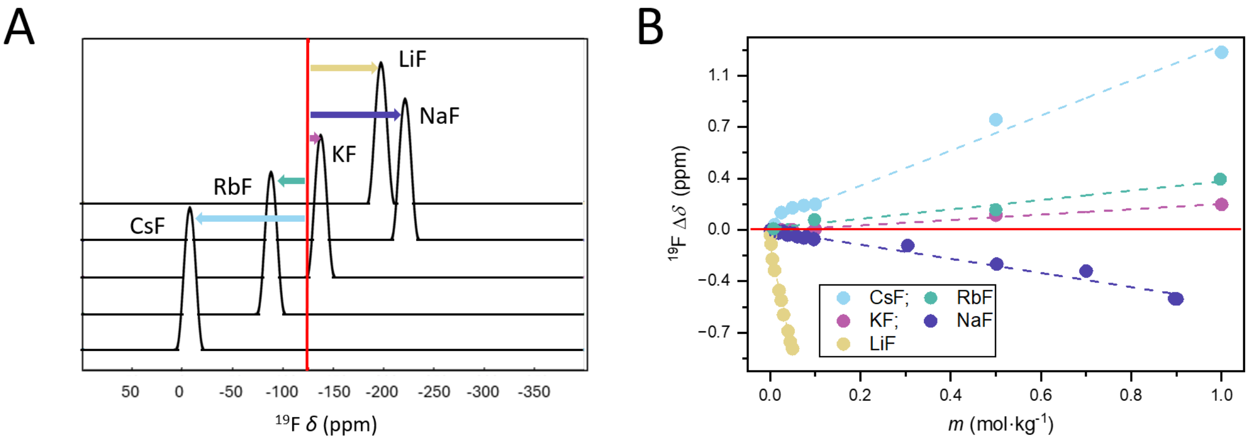

Scheme S1. Graphical representation of solid-state approach. **A.** Schematic representation of solid state ^{19}F spectra for XF (data from ref. S2) along with chemical shift for free ^{19}F at ~ 298 K. **B.** Changes of ^{19}F $\Delta\delta$ as function of molarity at ~ 298 K.

Table S1. The source and purity of chemicals used for this study. Chemicals were used without additional purification, except for deionized H_2O, which was prepared with a Millipore ultrapure water system (resistivity ≥18.0 MΩ·cm) and degassed prior to use.

Chemical Name	Source	Supplier's Purity Estimate
Sodium fluoride (NaF)	Sigma Aldrich	99.99%
Lithium fluoride (LiF)	Sigma Aldrich	99.99%
Potassium fluoride (KF)	Sigma Aldrich	99.9%
Rubidium fluoride (RbF)	Sigma Aldrich	99.8%
Cesium fluoride (CsF)	Sigma Aldrich	99.99%
Tetramethyl ammonium fluoride (TMAF)	Sigma Aldrich	98%
sodium 2,2-dimethyl-2-silapentane-5-sulfonate-D_6 (DSS)	Cambridge Isotope Laboratories	98% (98% isotopic enrichment)
deuterated water (D_2O)	Cambridge Isotope Laboratories	99.999% isotopic enrichment
methanol-D_4 (CD_3OD)	Bruker	99.5% (99.8% isotopic enrichment)
80% ethylene glycol + 20% dimethyl sulfoxide-D_6 (DMSO-D_6)	Cambridge Isotope Laboratories	98% (99.9% isotopic enrichment)

Table S2. The molalities of XF solutions used in this study. Stock solutions of LiF, NaF, KF, RbF, and CsF were prepared and serial dilution was used to achieve desired concentrations.

Stock solution	Diluted Molalities
LiF, 0.05 mol·kg^{-1}	0.045, 0.04, 0.03, 0.02, 0.01, 0.005, 0.003, 0.001, 0.0005 mol·kg^{-1}
NaF, 0.917 mol·kg^{-1}	0.713, 0.511, 0.31, 0.097, 0.08, 0.06, 0.05, 0.0485, 0.04, 0.0388, 0.0291, 0.0194, 0.012, 0.0097, 0.001 mol·kg^{-1}
KF, 1.014 mol·kg^{-1}	0.504, 0.05, 0.001 mol·kg^{-1}
RbF, 1.060 mol·kg^{-1}	0.509, 0.0102, 0.001 mol·kg^{-1}
CsF, 1.011 mol·kg^{-1}	0.507, 0.101, 0.075, 0.05, 0.025, 0.01, 0.001 mol·kg^{-1}

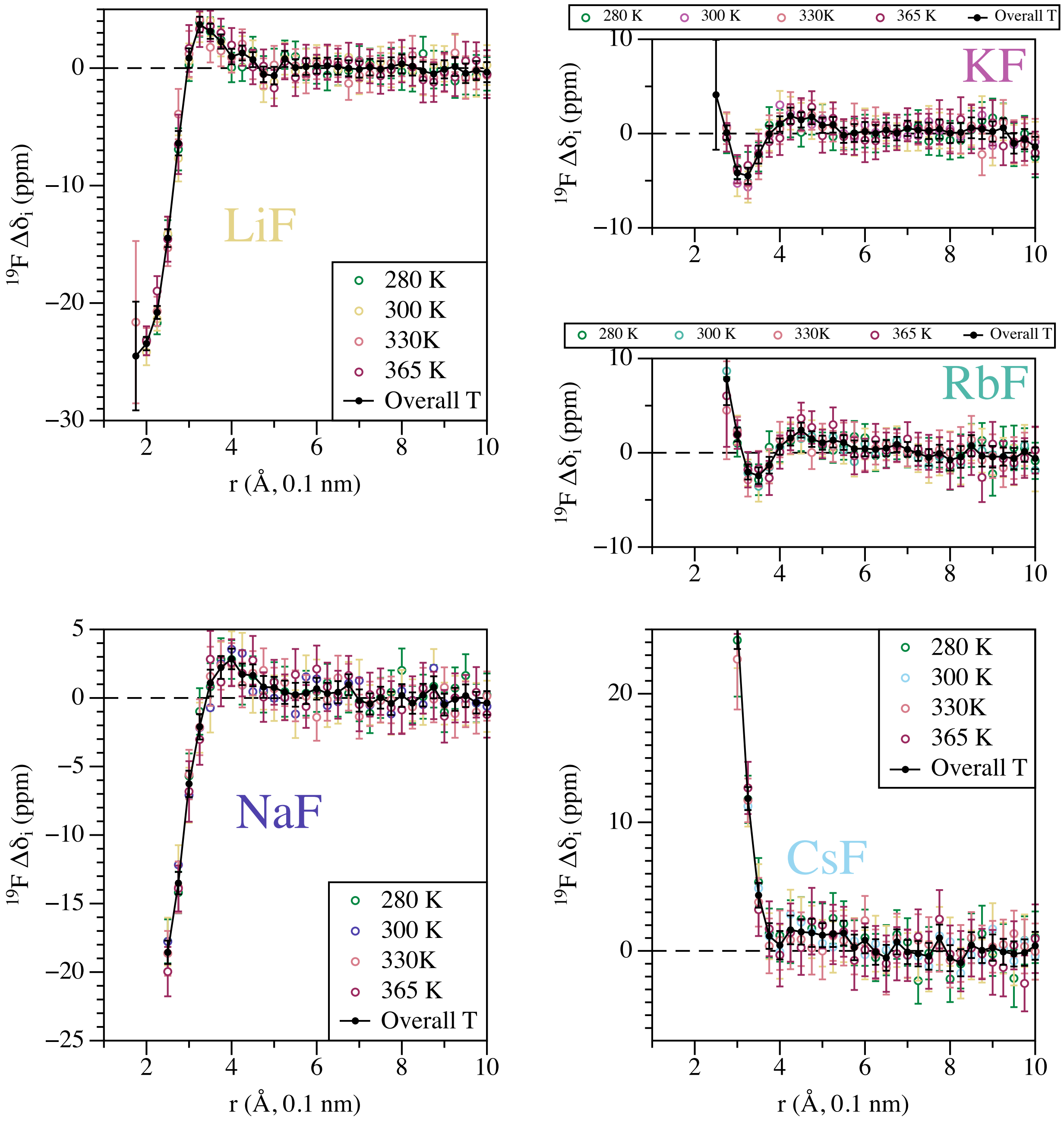

Figure S1. Ion-pair NMR ^{19}F chemical shift profile ($\boldsymbol{\Delta\delta_i}$) for each XF salt calculated independently for all temperatures along with the overall average that is reported in Fig. 2B of the main text.

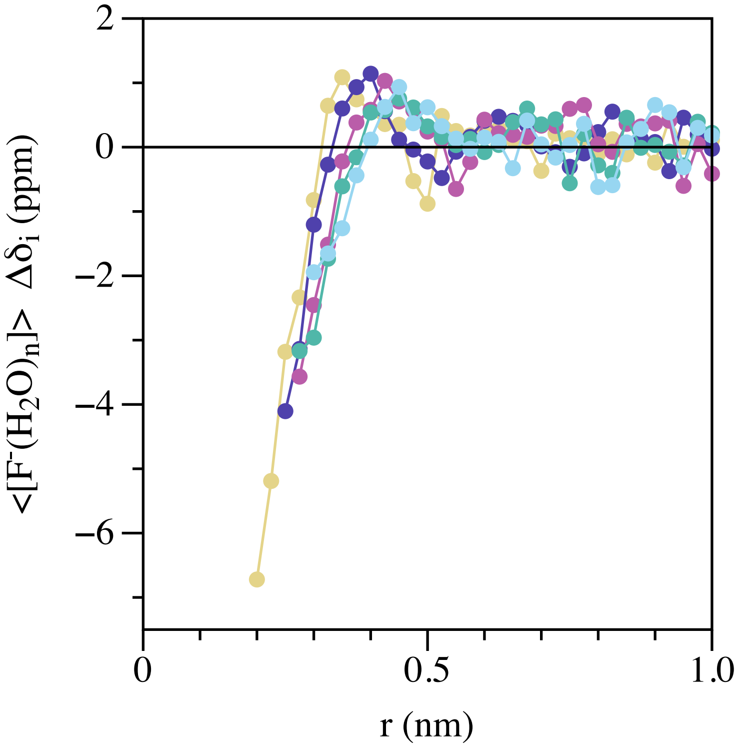

Figure S2 - Ion-pair ^{19}F NMR chemical shift profile calculated using the linear model that relates **FI-δ** to the number of first-shell solvent water molecules (**$^{19}F\Delta\delta_i$** = -18.1511 + 5.58846·n, Fig. 1D). The average number of water molecules in the first shell of F^- is binned in 0.05 nm intervals across all temperatures. Errors are not estimated.

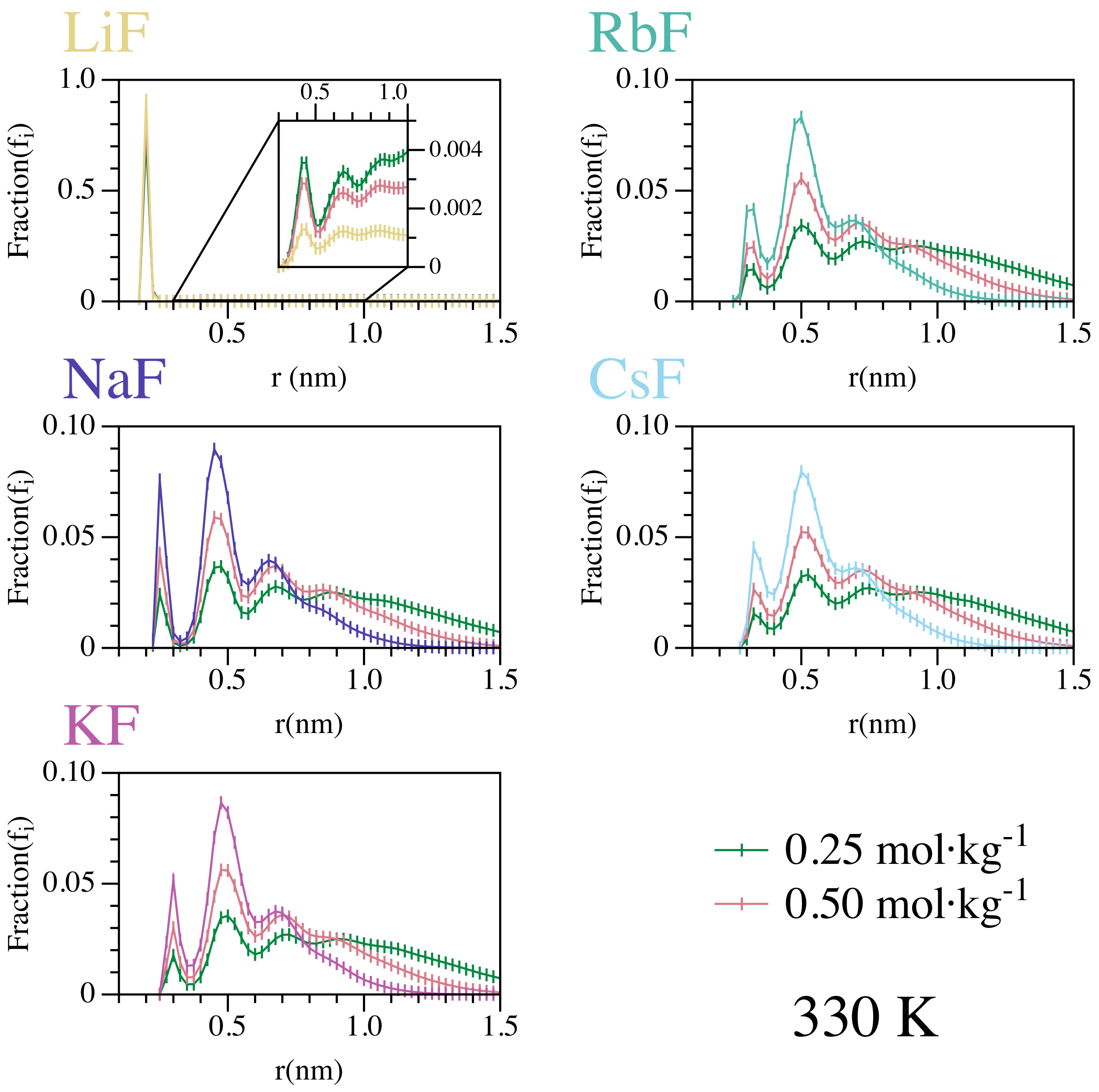

Figure S3. Minimum-distance distributions for each XF salt as a function of distance at varying molalities (0.25 mol·kg^{-1}, 0.5 mol·kg^{-1}, and 1.0 mol·kg^{-1}) and a fixed temperature (330 K). The distributions were calculated in 0.025 nm increments by counting the minimum distance between each ion and its nearest counterion and normalized to unity. The color coding is consistent across all panels, with the 1 mol·kg^{-1} molality shown in the same color as the XF salt label. The large CIP-region value for LiF is due to salt clustering (Fig. 2C), consistent with its low solubility. The temperature dependence of the CIP population is discussed in the main text and shown in the SI.

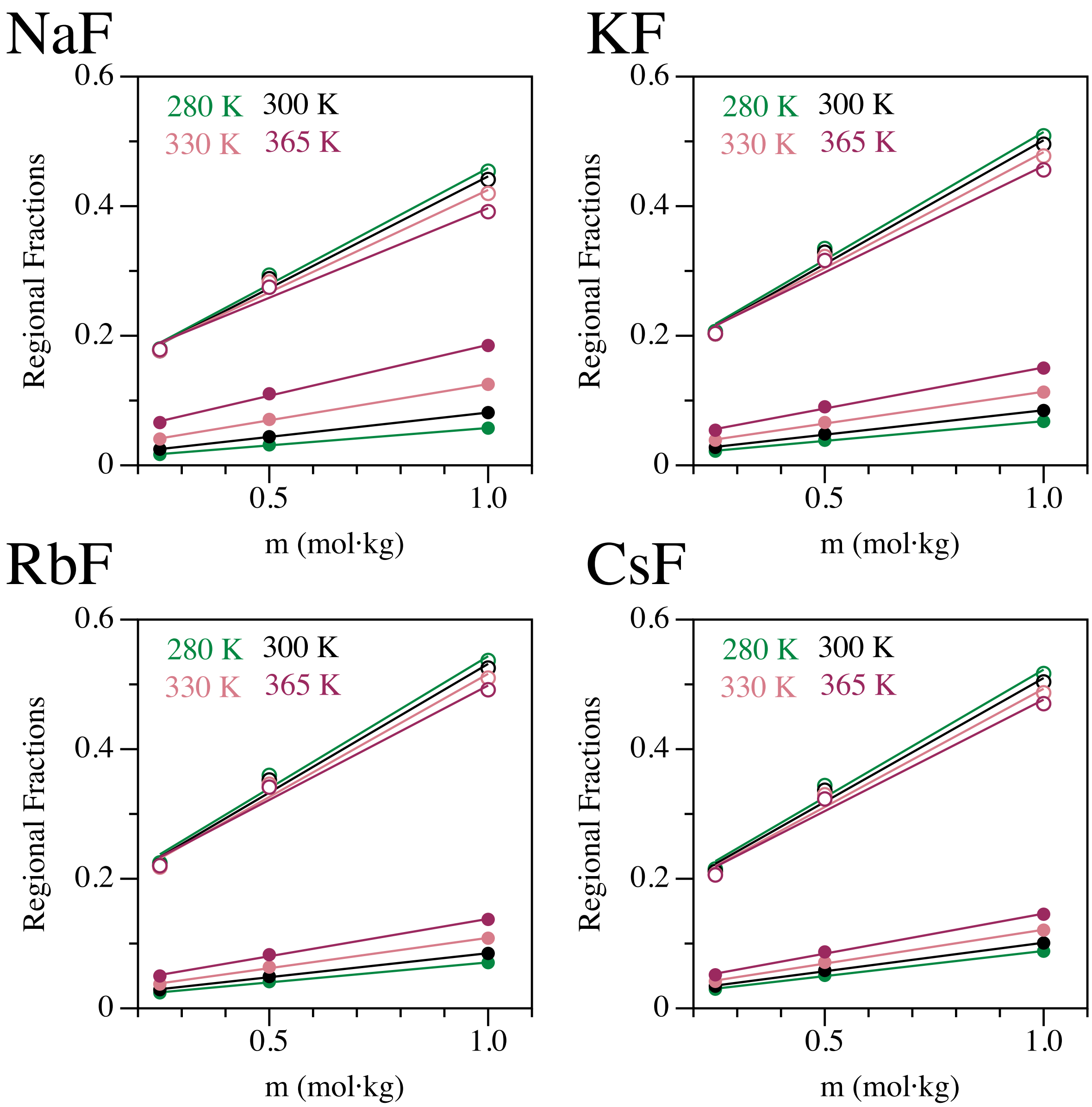

Figure S4. Regional populations for CIP (filled circles) and SIP (unfilled circles) calculated from the minimum distance distributions as a function of molality and temperature. The CIP and SIP regions were defined with cutoffs determined from RDF minima: NaF: [3.2, 5.5], KF: [3.6, 6], RbF:[3.7, 6.1], CsF: [3.9, 6.2]. The population of CIP and SIP increases with molality. At a given molality, the CIP and SIP populations change in opposite directions: while the CIP population increases with temperature, the SIP populations drop slightly.

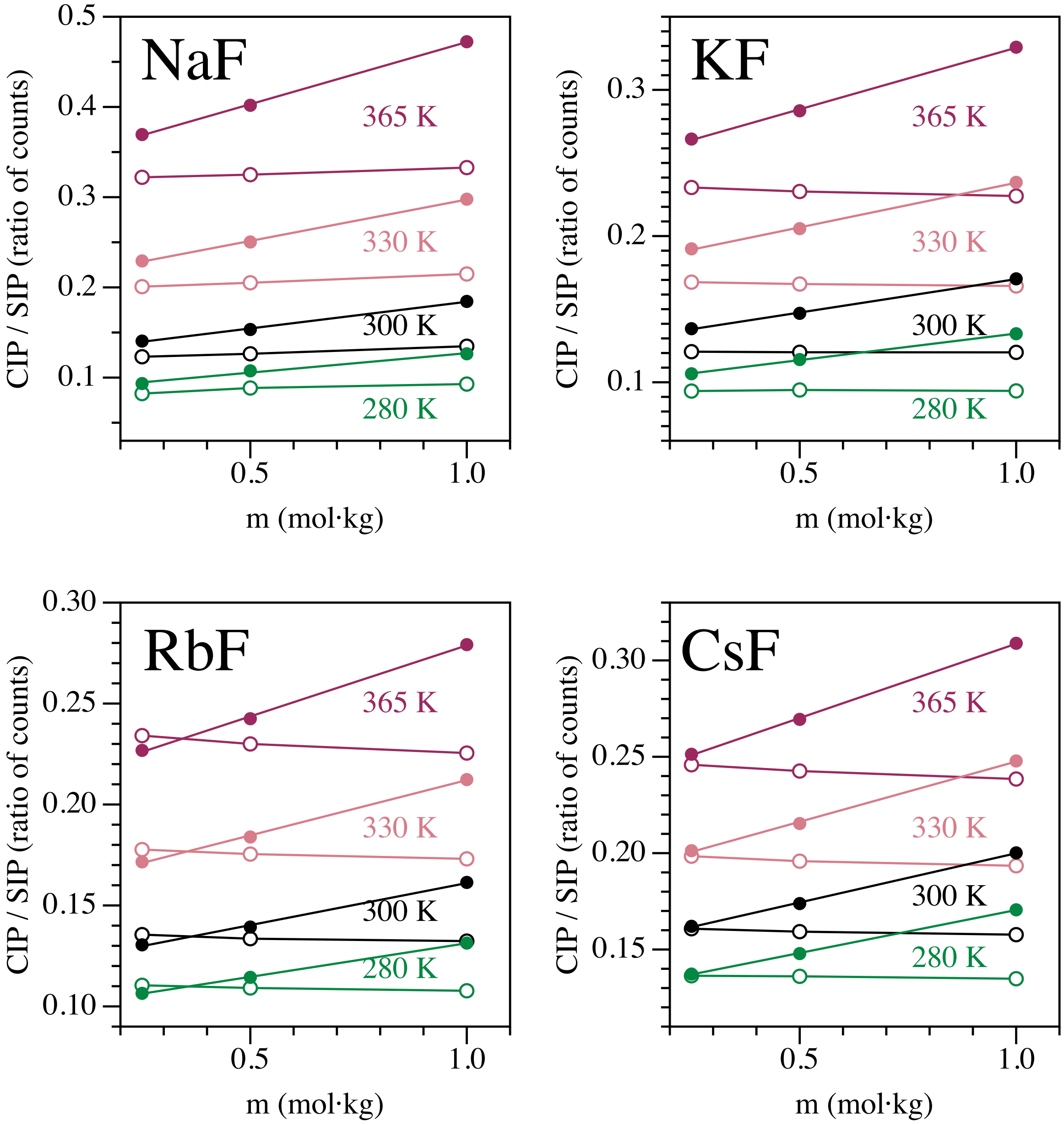

Figure S5. Ratio of CIP to SIP populations, as a function of molality, calculated from radial (unfilled circles) and minimum distance (filled circles) distributions for NaF through CsF. The ratio is flat for RDF. The relative population of CIP to SIP is flat for RDF and increases with molality for the minimum distance distribution. The increase in the minimum-distance distribution ratio with molality is due to the exclusion of SIP interactions that have already been consumed by CIP pairs. Consequently, we use the RDF ratio when estimating the chemical shift using the equilibrium constant for low-molality solutions.

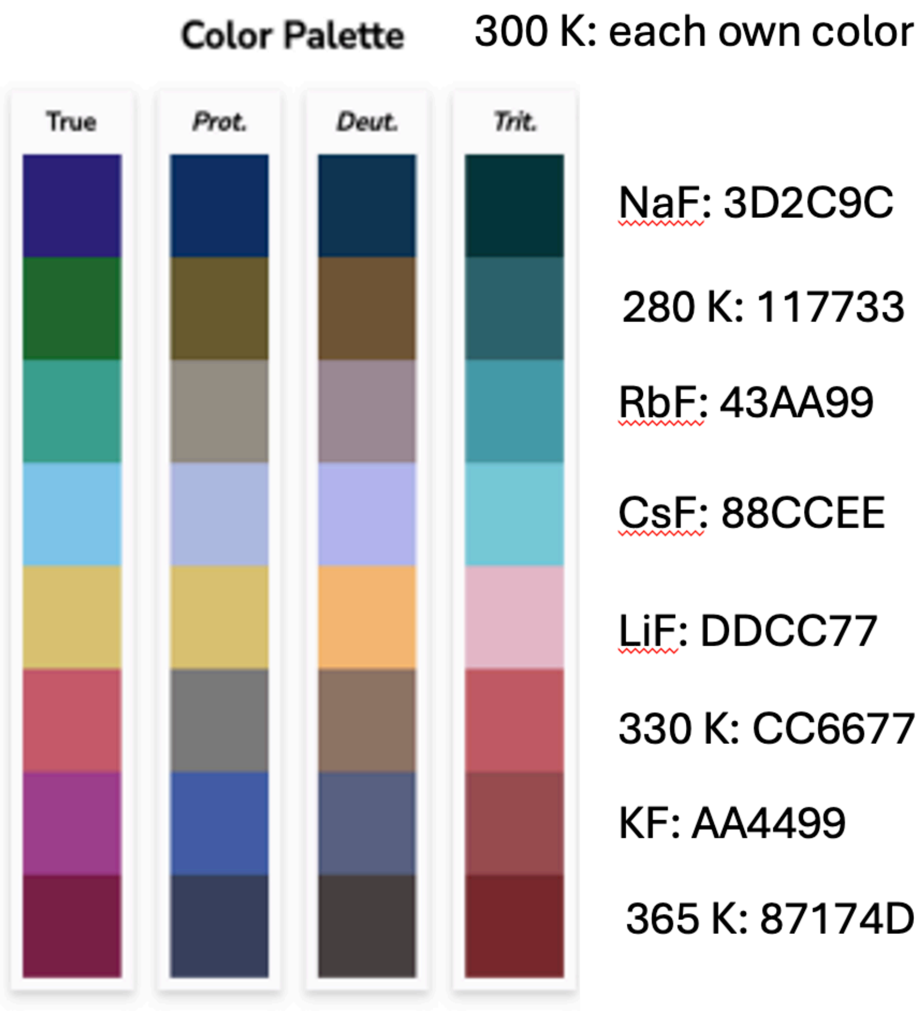

Figure S6. Color palette used throughout for XF salts, temperature, and molality. Citation for color palette: Nichols, D. (a). Coloring for Colorblindness. https://davidmathlogic.com/colorblind/#%23332288-%23117733-%2344AA99-%2388CCEE-%23DDCC77-%23CC6677-%23AA4499-%23882255

References

[S1] Deverell, C.; Schaumburg, K.; Bernstein, H. J. 19F Nuclear Magnetic Resonance Chemical Shift of Alkali Fluorides in Light- and Heavy-Water Solutions, *J. Chem. Phys.* **1968**, *49* (3), 1276-1283. DOI:10.1063/1.1670220

[S2] Sadoc, A.; Body, M.; Legein, C.; Biswal, M.; Fayon, F.; Rocquefelte, X.; Boucher, F. NMR parameters in alkali, alkaline earth and rare earth fluorides from first principle calculations. *Phys. Chem. Chem. Phys.* **2011**, *13* (41), 18539-18550. DOI: 10.1039/C1CP21253B
